# TOSQ: Transparent Object Segmentation via Query-Based Dictionary Lookup with Transformers

**DOI:** 10.3390/s25154700

**Published:** 2025-07-30

**Authors:** Bin Ma, Ming Ma, Ruiguang Li, Jiawei Zheng, Deping Li

**Affiliations:** 1 College of Electronic and Information Engineering, Hubei University of Automatic Technology, No. 167 Checheng West Road, Shiyan 442002, China; mab_dy@huat.edu.cn (B.M.);; 2 School of Intelligent Systems Science and Engineering, Jinan University, No. 206 Qianshan Road, Zhuhai 519070, China

**Keywords:** deeping learning, semantic segmentation of transparent objects, transformer, semantic segmentation

## Abstract

Sensing transparent objects has many applications in human daily life, including robot navigation and grasping. However, this task presents significant challenges due to the unpredictable nature of scenes that extend beyond/behind transparent objects, particularly the lack of fixed visual patterns and strong background interference. This paper aims to solve the transparent object segmentation problem by leveraging the intrinsic global modeling capabilities of transformer architectures. We design a Query Parsing Module (QPM) that innovatively formulates segmentation as a dictionary lookup problem, differing fundamentally from conventional pixel-wise mechanisms, e.g., via attention-based prototype matching, and a set of learnable class prototypes as query inputs. Based on QPM, we propose a high-performance transformer-based end-to-end segmentation model, Transparent Object Segmentation through Query (TOSQ). TOSQ’s encoder is based on the Segformer’s backbone, and its decoder consists of a series of QPM modules, which progressively refine segmentation masks by the proposed QPMs. TOSQ achieves state-of-the-art performance on the Trans10K-V2 dataset (76.63% mIoU, 95.34% Acc), with particularly significant gains in challenging categories like windows (+23.59%) and glass doors (+11.22%), demonstrating its superior capability in transparent object segmentation.

## 1. Introduction

Transparent objects, such as car glass, glass cups, plastic bottles, glass doors, and glass walls, are prevalent in daily life but pose unique segmentation challenges due to their lack of contrast with backgrounds and complex optical properties. Effective transparent object segmentation is critical for applications like autonomous driving, augmented reality, and robot navigation in environments with glass partitions.

Although significant progress has been made in collecting a large amount of image data containing transparent objects [1,2,3], segmenting transparent objects from a single 2D RGB image remains a huge challenge. Industrial deployment of RGB imaging faces inherent constraints, including limited spectral sensitivity (visible band only), depth ambiguity in object stacking scenarios, vulnerability to specular interference under harsh lighting, and insufficient material discriminability among transparent substrates. Depth maps (RGB-D) [4,5], thermal maps (RGB-T) [6], and polarization maps (RGB-P) [7,8] are typically used by traditional methods to identify transparent objects in images. Using additional data can indeed help the model better understand the features of transparent objects and improve the accuracy of transparent object segmentation, but this data is often difficult to obtain.

In addition, transparent objects remain challenging due to their lack of fixed textures and strong dependency on background scenes. This motivates us to propose a dictionary-based formulation that explicitly models transparent attributes through learnable prototypes. To address the challenge of transparent object segmentation in single RGB images, we propose an efficient Query Parsing Module (QPM) and a high-performance transformer-based model, TOSQ. Our key contributions are:

(1) An end-to-end Transformer architecture for transparent objects. We propose TOSQ, a fully transformer-based model specifically designed for segmenting transparent objects in RGB images. By leveraging the intrinsic global modeling capability of Transformers, TOSQ effectively handles the ambiguous boundaries and variable appearances caused by light refraction/reflection.

(2) Novel dictionary-formulated segmentation paradigm. We design QPM to reformulate semantic segmentation as a dictionary look-up problem, where transparent objects are represented as compositions of learnable prototypes. A set of class-aware prototypes is introduced as dynamic query inputs, enabling the model to adaptively capture transparent object features (e.g., edge distortions, specular highlights) without manual feature engineering.

(3) State-of-the-art performance. Extensive experiments on the Trans10K-V2 dataset [2] demonstrate that TOSQ achieves state-of-the-art performance, with 76.63% mIoU and 95.34% accuracy, outperforming both convolutional-neural-network-based and transformer-based methods.

## 2. Related Work

Transparent object segmentation is an essential task in robot perception that classifies each pixel value of an image as transparent or non-transparent. This task enables autonomous robots to navigate in unknown environments, such as laboratories, markets, or factories, without colliding with glass walls or windows. Moreover, transparent object segmentation is a fundamental technique for other transparent object perception tasks, such as object pose estimation. In this section, we provide an overview of single-image transparent object segmentation methods developed over the past decade.

### 2.1. Hand-Crafted Feature-Based Methods

In early studies on transparent object segmentation, visual cues such as boundary features and strong highlights on the surface were mainly used to predict the regions of transparent objects [9,10]. McHenry et al. [9] presented a region-based segmentation method for detecting objects made of transparent materials such as glass. They combine two complementary measures of affinity between regions made of the same material and discrepancy between regions made of different ones into a single objective function and use the geodesic active contour framework to minimize this function over pixel labels. However, these methods only work well under the strong assumption that the background is similar on both sides of all glass edges.

### 2.2. CNN-Based Methods

In recent years, convolutional neural networks have seen extensive use by researchers for transparent object segmentation [11,12,13]. Chen et al. [14] introduced TOM-Net, a U-shaped method with consistent feature space dimensions in both encoder and decoder layers. Madessa et al. [15] employed Mask R-CNN to detect individual transparent objects. Mei et al. [16] proposed GDNet, which utilized a large-field contextual feature integration module and convolutional block attention module (CBAM) for feature fusion. Xu et al. [17] utilized dense connections between different atrous convolution blocks to restore detailed information for glass segmentation. Ref. [18] used multiple Discriminability Enhancement (DE) modules and Focus-and-Exploration-Based Fusion (FEBF) for coarse-to-fine glass segmentation. Xie et al. [3] introduced TransLab, a boundary-aware segmentation method that leverages boundaries to enhance segmentation performance. Cao et al. [19] proposed a boundary-aware segmentation method with an adaptive ASPP module capturing features of multiple receptive fields. However, edge supervision [3,19] may limit the generality of learning objects with various shapes. To address this, He et al. [20] introduced EBLNet, which utilizes an edge-aware point-based graph convolution network module for enhanced boundary prediction. Lin et al. [21] utilized a Rich Context Aggregation Module (RCAM) to extract multi-scale boundary features and a reflection-based refinement module for differentiating glass regions from non-glass regions. Finally, Lin et al. [22] proposed a method for glass surface detection by integrating contextual relationships of scenes with spatial information, differing from other works that focus on low-level feature extraction, such as boundary and reflections.

### 2.3. Transformer-Based Methods

In recent years, Transformer has been successfully applied in both high-level vision and low-level vision, including transparent object segmentation [23,24]. Xie et al. [2] developed a transformer-based network (Trans2Seg) for transparent object segmentation with a transformer encoder–decoder architecture. The transformer encoder in Trans2Seg offers a crucial global receptive field for transparent objects segmentation. Meanwhile, the transformer decoder adopts a dictionary look-up approach with a series of learnable queries, where each query represents a distinct object category. Zhang et al. [25] propose Trans4Trans, an architecture utilizing a transformer-based encoder and decoder, featuring a dual-head design to address transparent object segmentation, and incorporating a Transformer Parsing Module to integrate multi-scale representations. Trans2Seg and our proposed method share similar components in the pipeline, including Transformer encoder and decoder, and a set of learnable class prototypes as the query. The biggest difference is the learnable class prototype interaction with the feature map. Trans2Seg uses a CNN-Transformer hybrid backbone network as an encoder to generate feature maps with a single resolution and interacts with each class prototype using the feature maps generated by the last encoder module. Our proposed method adopts a pure transformer architecture encoder to generate multi-level feature maps with resolution differences, and each class prototype interacts with the hierarchical feature maps output by each encoder module.

## 3. Our Method

**Figure 1:**
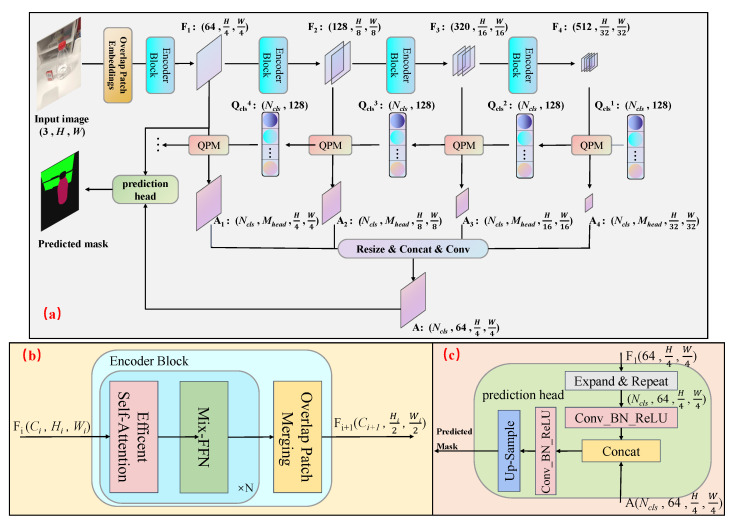
The overview of the TOSQ model. (**a**) The architecture of TOSQ. (**b**) The hierarchical Transformer encoder. (**c**) The lightweight small prediction head. Notations: *F* (feature map), *H* (height of the feature map), *W* (width of the feature map), $Q_{cls}$ (query class prototypes), $N_{cls}$ (number of categories), *A* (attention map), *C* (channel dimension). Operations: Concat (concatenation), Conv (convolution), Up-sample (upsampling). Component: Lightweight Small Prediction Head (a simplified prediction module designed to reduce computational overhead while maintaining accuracy), QPM: Query Parsing Module, details as seen in Section Decoder, Mix-FFN: replaces the standard FFN (Feed Forward Network) with a 3×3 depthwise convolution between two linear layers, leveraging zero-padding to encode positional information implicitly and eliminating the need for explicit position encodings.

This section introduces the TOSQ model, which draws upon and refines the ideas of transparent object semantic segmentation methods. As shown in Figure 1, the TOSQ model is based on the transformer architecture model. The TOSQ model includes a multi-scale hierarchical Transformer encoder, a decoder composed of several query parsing modules QPM (Query Parsing Module), and a lightweight small prediction head. Given an image with size 3×H×W, we first divide it into patches and use these patches as input to the hierarchical Transformer encoder to get multi-level features with resolution $\left(\frac{1}{4},\frac{1}{8},\frac{1}{16},\frac{1}{32}\right)$ of the original image. Then, the QPM module in the decoder combines the multi-level feature maps with a set of learnable class prototypes. These class prototypes have a size of $N_{cls}\times 128$, where $N_{cls}$ is the number of categories. This combination generates a new set of size-invariant but parsed class prototypes. The attention feature map of the input class prototype to the input feature map has a size of $\left(N_{cls},M_{head},H_{i},W_{i}\right)$, Here, $M_{head}$ is the number of attention heads in the multi-head cross-attention layer of the QPM module. The dimensions $H_{i}$ and $W_{i}$ represent the height and width of the feature map output by the encoder module, respectively. Specifically, $H_{i}$ and $W_{i}$ correspond to the resolutions $\left(\frac{H}{4},\frac{W}{4}\right)$, $\left(\frac{H}{8},\frac{W}{8}\right)$, $\left(\frac{H}{16},\frac{W}{16}\right)$ and $\left(\frac{H}{32},\frac{W}{32}\right)$ for the respective encoder outputs, ensuring consistency with Figure 1. Finally, the attention feature maps output by the QPM modules are fused, and the fused attention feature maps are fed into a lightweight prediction head to obtain the final prediction result. In the remainder of this section, we detail the proposed encoder and decoder designs.

### 3.1. Encoder

To obtain high-resolution weak semantic information feature maps to preserve the detailed information of object boundaries in the image and low-resolution strong semantic information feature maps to obtain the semantic category features of objects in the image, we adopt the MiT (Mix Transformer encoders) in the Segformer model [26] as the image feature encoder. The design of MiT is somewhat inspired by the ViT (Vision Transformer) [27] model and has been customized and optimized for semantic segmentation. Unlike ViT, which can only generate single-resolution feature maps, given an input image, MiT will generate multi-level features like convolutional neural networks. After each encoder module, the image resolution will be downsampled by a quarter, and the channel dimension will increase to maintain more information. The structure of each MiT encoder module is shown in Figure 1b. MiT, which can provide both high-resolution coarse-grained features and low-resolution fine-grained features, can effectively improve the performance of semantic segmentation. To achieve a balance between accuracy and speed, the TOSQ model proposed in this paper selects MiT-B2 as the encoder. The four encoder modules have 3, 4, 6, and 3 repetitions, respectively. The customized and efficient multi-head self-attention layer has 1, 2, 5, and 8 heads, respectively. In the original formulation, the query *Q*, key *K*, and value *V* matrices all share the dimension $N\times d_{\mathrm{head}}$, where N=H×W denotes the flattened spatial length, $d_{head}$ is the dimension of each head. Self-attention is then computed as

(1) $$\mathrm{Attention}\left(Q,K,V\right)=\mathrm{Softmax}\left(\frac{QK^{\;⊤}}{\sqrt{d_{\mathrm{head}}}}\right)V,$$

where the inner product $QK^{\;⊤}$ measures the pairwise similarity between queries and keys, and the resulting weights are applied to the values *V*.

To lower the quadratic complexity, MiT introduces a sequence-reduction factor *R*. The key tensor *K* is first reshaped to dimensions $\frac{N}{R}\times \left(C\;\cdot \;R\right)$, then projected back to *C* channels via a linear layer:

(2) $$\hat{K}=\mathrm{Reshape}\;\left(\frac{N}{R},\;C\;\cdot \;R\right)\left(K\right),\;K^{\prime }=\mathrm{Linear}\left(C\;\cdot \;R,\;C\right)\left(\hat{K}\right),$$

thereby reducing the self-attention complexity from $O(N^{2})$ to $O(N^{2}/R)$. Therefore, the new *K* has dimensions $\frac{N}{R}\times C$. As a result, the complexity of the self-attention mechanism is reduced from $O(N^{2})$ to $O(\frac{N^{2}}{R})$. In MiT-B2, the sequence scaling factors *R* for the four encoder modules are 8, 4, 2, and 1, respectively.

### 3.2. Decoder

The proposed TOSQ model employs a novel decoder architecture consisting of four sequential QPMs. Each QPM performs an adaptive fusion between the feature maps from corresponding encoder stages and a set of learnable class prototypes, generating two outputs: (1) refined class prototypes for subsequent modules, and (2) attention feature maps preserving the spatial dimensions of the input features.

The decoding process fundamentally reformulates semantic segmentation as a dynamic dictionary query problem. In this framework, the class prototypes, representing meta-features of target categories, serve as learnable queries. Encoder-derived feature maps are projected to aligned dimensions as keys and values. A multi-head cross-attention mechanism then establishes correspondence between queries (prototypes) and keys (image features).

The attention feature maps produced at each stage encode the spatial distribution of prototype matches, which are progressively aggregated to form the final segmentation mask. Simultaneously, the cross-attention operation outputs updated prototypes that incorporate image-specific adaptations. This iterative refinement enables context-aware prototype evolution across QPM stages, hierarchical feature integration from local patterns to global semantics, and adaptive adjustment to varying transparent object characteristics.

The complete architecture effectively transforms transparent object segmentation into a multi-scale dictionary lookup process, where prototypes dynamically adapt to input-specific visual patterns through successive refinement stages.

In detail, due to the significant influence of the external environment on the texture of different types of transparent objects, the details between classes are very similar and can only be distinguished by shape based on the environment. Therefore, explicitly incorporating category information into the model can help the model correctly distinguish the types of transparent objects. Therefore, we design a set of parameter learnable class prototypes as one of the inputs for the decoder in the TOSQ model, with a size of $N_{cls}\times C_{hilden}$, where $N_{cls}$ is the number of categories in the dataset, and $C_{hilden}$ is the hidden dimension of each class tensor. Due to limited computing resources and a trade-off between speed and accuracy, the hidden dimension selection for each class of the learnable class prototype in the SQT model proposed in this paper is 128.

**Figure 2:**
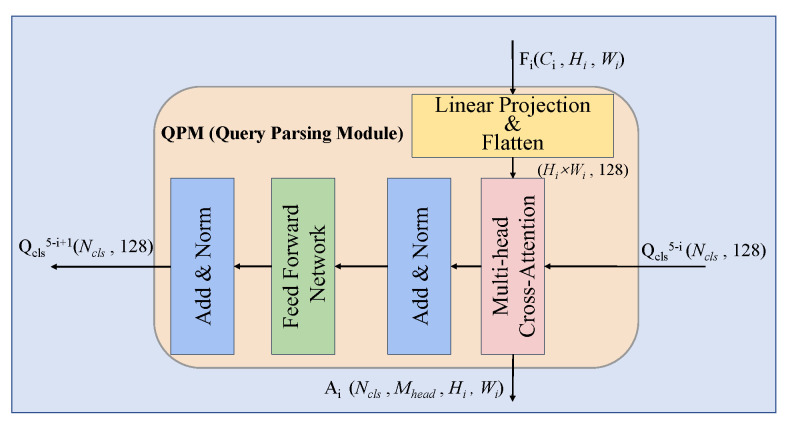
The internal architecture of the QPM module.

The internal details of the QPM module are shown in Figure 2. The feature map $F_{i}$ output from the *i*-th encoder module is first subjected to a layer of linear transformation, aligning the size of the channel dimension with the size of the hidden dimension of the class prototype $Q_{cls}^{5-i}$. In order to enable cross attention between the feature map $F_{i}$ and the class prototype $Q_{cls}^{5-i}$, after dimension alignment, the new feature map $F_{i}^{\prime }$ is flattened to a size of $\left(128,H_{i},W_{i}\right)$ and becomes $\left(H_{i}\times W_{i},128\right)$. Next, in order to model and refine the interior of each class prototype tensor, the operation results of the multi-head cross-attention layer are input into a feedforward linear layer FFN after residual connection and layer normalization. This layer contains two fully connected layers, the first fully connected layer magnifies the input hidden dimension by four times, and the second fully connected layer scales the hidden dimension back. Finally, after residual connections and layer normalization, the output from FFN yields a new class prototype $Q_{cls}^{5-i+1}$ that has been parsed by the QPM module and feature maps.

After the input image is processed by the encoder and decoder, a series of feature maps and attention feature maps are obtained. To obtain the final prediction mask, this paper also designs a small, lightweight prediction head, whose internal architecture is shown in Figure 1c. To provide more detailed boundary contours of transparent objects in the final prediction result, the input of this small prediction head includes not only the output result *A* of the query parsing module QPM but also the high-resolution low semantic information feature map $F_{1}$ output by the first encoder module in the encoder. The feature map output by the first encoder module has not undergone much deep modeling, and the resolution is also high, retaining a lot of detailed information about the target category. Participating in the calculation of the prediction mask together can help obtain more accurate prediction mask contours, which can effectively improve the accuracy of semantic segmentation results. Before concatenating feature map $F_{1}$ and attention feature map *A*, it is necessary to align their dimensions. The feature map $F_{1}$ output from the decoder module will first be expanded in the first dimension within the prediction head, meaning its dimension size will increase from $64\times \frac{H}{4}\times \frac{W}{4}$ and be extended to $1\times 64\times \frac{H}{4}\times \frac{W}{4}$; then, the first dimension is repeated $N_{cls}$ times, and the dimension size becomes $N_{cls}\times 64\times \frac{H}{4}\times \frac{W}{4}$. Then, it sends the physically aligned feature map $F_{1}$ into a convolution kernel with a 3×3 convolutional layer for semantic space modeling and alignment after the batchnormalization [28] and ReLU activation function [29]. Then, the result of concatenating feature map $F_{1}$ with attention feature map *A* is also sent to a Conv-BN-ReLU module and then upsampled to obtain the final prediction mask.

## 4. Experiments

### 4.1. Details

**Figure 3:**
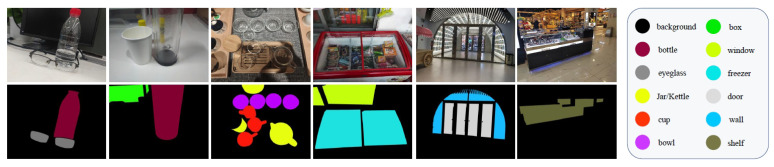
Images in Trans10K-v2 dataset [2]. The first row shows sample images and the second shows the segmentation masks. The color scheme that encodes the object categories is listed on the right of the figure. Zoom in for the best view.

We used the mmsegmentation open source code framework [30] based on Pytorch to implement the TOSQ model and conducted experiments on the Trans10Kv2 dataset [2], as shown in Figure 3. The Trans10Kv2 dataset contains a total of 10,428 images, including 11 transparent object categories: shelves, jars, freezers, windows, glass doors, glasses, cups, glass walls, glass bowls, water bottles, and boxes. Among them, 5000 images are used as the training set, 1000 images are used as the validation set, and 4428 images are used as the test set. In addition, the TOSQ model experiment proposed in this article uses an Adam optimizer with an epsilon parameter of $1\times 10^{-8}$ and weight decay coefficient of $5\times 10^{-3}$. The sample size for each batch is 8, the total batch iteration is 160 lk, and the experiment is conducted using 1 NVDIA Geforce RTX 3090 (NVIDIA Corporation, Santa Clara, CA, USA). The learning rate is set to $1.5\times 10^{-5}$ and is attenuated using a Poly strategy with parameter of 0.9. In order to ensure stable convergence of the model, a learning rate warm-up was also used, allowing the learning rate to linearly increase from the initial learning rate of one millionth to the present value within 1.6 k iteration steps, and then attenuated. Although the TOSQ model does not require scaling the image to a fixed size due to position encoding, in order to fairly compare with models such as Trans2Seg [2] and Trans4Trans [25], the image is randomly scaled and randomly cropped to a resolution of 512×512 during training. During testing, the image is uniformly scaled to a resolution of 512×512, and the aspect ratio of the image is maintained during scaling. The remaining portion is filled with 0, and the filled portion is not included in the loss calculation and prediction.

Performance was evaluated using three standard semantic segmentation metrics: Pixel Accuracy (Acc), Mean IoU (mIoU), and Category IoU.

### 4.2. Comparisons with State-of-the-Art Models

As shown in Table 1, TOSQ achieves state-of-the-art performance on the Trans10K-V2 benchmark with 76.63% mIoU and 95.34% accuracy, outperforming previous transformer-based methods like Trans4Trans by +1.49% mIoU and +0.33% accuracy while maintaining similar computational efficiency.

The performance gains are particularly pronounced in challenging categories where transparent objects exhibit visual ambiguity. (1) Structural elements: windows (+23.59% mIoU) and glass doors (+11.22%); (2) thin-walled containers: glass bowls (+10.05%), cups (+2.48%), and cans (+7.48%). This advancement stems from two key innovations in our architecture. First, the QPM enables dynamic feature adaptation through prototype-based dictionary lookup. Second, learnable class prototypes explicitly encode category-specific priors for long-range context modeling (critical for large transparent surfaces) and fine-grained semantic discrimination (essential for similar categories).

The consistent improvements across all metrics validate TOSQ’s effectiveness in handling the unique challenges of transparent object segmentation, particularly in scenarios requiring optical distortion compensation, inter-class similarity resolution, and structural detail preservation.

### 4.3. Ablation Study

#### 4.3.1. Ablation Study on Encoder

Since one of our critical designs lies in a hierarchical Transformer encoder, we now analyze the effect of the encoder block of TOSQ, as shown in Table 2. It can be seen that as the number of encoder blocks increases and the model parameters increase, the Acc and mIoU of transparent objects show a significant increase. Especially when the number of encoder blocks increases from three to four, Acc increases from 85.86% to 95.34%, and mIoU increases from 38.90% to 76.63%. This indicates that advanced semantic information is crucial for transparent object segmentation.

#### 4.3.2. Ablation Study on the Class Prototypes

The learnable class prototype serves as a cornerstone of TOSQ’s architecture. To investigate its design impact, we conduct systematic experiments evaluating how the prototype dimension (D) affects segmentation performance (Table 3). Through experiments, expanding the hidden dimension from D = 128 to D = 256 yields consistent metric improvements: Acc increases from 95.34% to 95.53%, and mIoU increases from 76.63% to 77.47%. This performance gain demonstrates: the prototypes’ capacity to encode richer feature representations at higher dimensions and the effectiveness of dimension scaling for both global and class-wise metrics. However, considering computational trade-off between speed and accuracy, the hidden dimension selection for each class of the learnable class prototype in the TOSQ model is set to 128.

### 4.4. Cross-Dataset Generalization on Cityscapes

To evaluate the generalization capability of TOSQ beyond transparent objects, we further tested it on the Cityscapes urban-scene dataset. All models (TOSQ and Trans4Trans) were evaluated under identical experimental protocols: both used a 512×512 input resolution, a MIT-B2 backbone pre-trained on ImageNet, and were trained for 160 k iterations with a batch size of 2 (training) and 1 (testing). Optimization was performed using Adam ($\varepsilon =1\times 10^{8}$, weight decay $=5\times 10^{-3}$) with a learning rate of $1.5\times 10^{-5}$, which was decayed using a polynomial schedule (power = 0.9).

Table 4 shows that TOSQ achieves higher mIoU (+3.0%) and Pixel Accuracy (+1.0%) than Trans4Trans, demonstrating its robustness to domain shifts (transparent objects → urban scenes). Notably, TOSQ excels in fine-grained categories such as traffic signs (+6.5%), vegetation (+0.3%), and person (+0.9%), validating the effectiveness of its learnable class prototypes for semantic discrimination.

## 5. Conclusions

In this paper, we introduce a novel approach to the semantic segmentation of transparent objects by leveraging a Transformer-based model, TOSQ, which incorporates the innovative QPMs. The TOSQ model’s encoder, built upon the MiT backbone, provides a global receptive field and generates hierarchical multi-resolution feature maps, significantly enhancing the model’s performance in transparent object segmentation. The decoder, composed of multiple QPMs, reformulates the segmentation task as a dictionary query problem using learnable class prototypes, effectively improving the model’s ability to distinguish between similar categories. Experimental results on the Trans10K-V2 dataset demonstrate that TOSQ achieves state-of-the-art performance, with 76.63% mIoU and 95.34% accuracy, outperforming both convolutional neural network-based and other transformer-based methods. Furthermore, TOSQ achieves these results with a computational efficiency of only 41.48 GFLOPs, highlighting its effectiveness and efficiency for real-world applications. TOSQ’s RGB-only design struggles with variable lighting, transparent/specular surfaces, and industrial safety requirements (≤0.1% error, ≥30 FPS). Future work should integrate multimodal inputs (e.g., polarization, LiDAR) and uncertainty estimation, validated on industrial datasets (e.g., MVTec AD).

## Figures and Tables

**Table 1:** Computation complexity in GFLOPs and category-wise accuracy evaluation and comparison with state-of-the-art semantic segmentation methods on the Trans10K-v2 dataset. Bold numbers indicate the best performance among all compared methods.

Method	GFLOPs	ACC	mIoU	Category IoU											
				Background	Shelf	Jar/Tank	Freezer	Window	Door	Eyeglass	Cup	Wall	Bowl	Bottle	Box
FPENet [31]	0.76	70.31	10.14	74.97	0.01	0.00	0.02	2.11	2.83	0.00	16.84	24.81	0.00	0.04	0.00
ESPNetv2 [32]	0.83	73.03	12.27	78.98	0.00	0.00	0.00	0.00	6.17	0.00	30.65	37.03	0.00	0.00	0.00
ContextNet [33]	0.87	86.75	46.69	89.86	23.22	34.88	32.34	44.24	42.25	50.36	65.23	60.00	43.88	53.81	20.17
FastSCNN [34]	1.01	88.05	51.93	90.64	32.76	41.12	47.28	47.47	44.64	48.99	67.88	63.80	55.08	58.86	24.65
DFANet [35]	1.02	85.15	42.54	88.49	26.65	27.84	28.94	46.27	39.47	33.06	58.87	59.45	43.22	44.87	13.37
ENet [36]	2.09	71.67	8.50	79.74	0.00	0.00	0.00	0.00	0.00	0.00	0.00	22.25	0.00	0.00	0.00
HRNet w18 [37]	4.20	89.58	54.25	92.47	27.66	45.08	40.53	45.66	45.00	68.05	73.24	64.86	52.85	62.52	33.02
HardNet [38]	4.42	90.19	56.19	92.87	34.62	47.50	42.40	49.78	49.19	62.33	72.93	68.32	58.14	65.33	30.90
DABNet [39]	5.18	77.43	15.27	81.19	0.00	0.09	0.00	4.10	10.49	0.00	36.18	42.83	0.00	8.30	0.00
LEDNet [40]	6.23	86.07	46.40	88.59	28.13	36.72	32.45	43.77	38.55	41.51	64.19	60.05	42.40	53.12	27.29
ICNet [28]	10.64	78.23	23.39	83.29	2.96	4.91	9.3	19.24	15.35	24.11	44.54	41.49	7.58	27.47	3.80
BiSeNet [36]	19.91	89.13	58.40	90.12	39.54	53.71	50.90	46.95	44.68	64.32	72.86	63.57	61.38	67.88	44.85
Trans4Trans-S [25]	19.92	94.57	74.15	95.60	**57.05**	71.18	**70.21**	63.95	61.25	81.67	87.34	78.52	77.13	81.00	64.88
Trans4Trans M [25]	34.38	95.01	75.14	96.08	55.81	71.46	69.25	65.16	63.96	83.84	88.21	**80.29**	76.33	**83.09**	**68.09**
DenseASPP [29]	36.20	90.86	63.01	91.39	42.41	60.93	64.75	48.97	51.40	65.72	75.64	67.93	67.03	70.26	49.64
DeepLabv3 [41]	37.98	92.75	68.87	93.82	51.29	64.65	65.71	55.26	57.19	77.06	81.89	72.64	70.81	77.44	58.63
FCN [42]	42.23	91.65	62.75	93.62	38.84	56.05	58.76	46.91	50.74	82.56	78.71	68.78	57.87	73.66	46.54
OCNet [43]	43.31	92.03	66.31	93.12	41.47	63.54	60.05	54.10	51.01	79.57	81.95	69.40	68.44	78.41	54.65
RefineNet [44]	44.56	87.99	58.18	90.63	30.62	53.17	55.95	42.72	46.59	70.82	76.01	62.91	57.05	70.34	41.32
Trans2Seg [2]	49.03	94.14	72.15	95.35	53.43	67.82	64.20	59.64	60.56	**88.52**	86.67	75.99	73.98	82.43	57.17
Translab [3]	61.31	92.67	69.00	93.90	54.36	64.48	65.14	54.58	57.72	79.85	81.61	72.82	69.63	77.50	56.43
DUNet [45]	123.69	90.67	59.01	93.07	34.20	50.95	54.96	43.19	45.05	79.80	76.07	65.29	54.33	68.57	42.64
UNet [46]	124.55	81.90	29.23	86.34	8.76	15.18	19.02	27.13	24.73	17.26	53.40	47.36	11.97	37.79	1.77
DANet [47]	198.00	92.70	68.81	93.69	47.69	66.05	70.18	53.01	56.15	77.73	82.89	72.24	72.18	77.87	56.06
PSPNe [48]	187.03	92.47	68.23	93.62	50.33	64.24	70.19	51.51	55.27	79.27	81.93	71.95	68.91	77.13	54.43
TOSQ-128	41.48	95.34	76.63	96.07	47.56	78.94	66.63	**88.75**	75.18	69.13	90.69	72.96	87.18	81.47	64.98
TOSQ-256	43.15	**95.53**	**77.47**	**96.22**	52.08	**81.82**	66.65	88.70	**75.69**	69.14	**91.03**	68.38	**87.82**	81.76	67.32

**Table 2:** Results of the ablation study on encoder. F1-2 represents only two feature maps in the encoder, F1 and F2. F1-3 represents three feature maps in the encoder—F1, F2 and F3. F1-4 represents four feature maps in the encoder—F1, F2, F3 and F4.

Layer	MParams	GFLOPs	Acc (%)	mIoU (%)
F1-2	3.484	27.58	70.63	24.82
F1-3	14.04	37.43	85.86	38.90
F1-4	25.31	41.48	95.34	76.63

**Table 3:** Results of the ablation study on the dimension of the learnable class prototypes. The size of learnable class prototypes is $N_{cls}\times C_{hilden}$, $N_{cls}$ is the number of categories in the dataset, and $C_{hilden}$ is the hidden dimension of each class tensor.

$C_{\mathrm{hilden}}$	MParams	GFLOPs	Acc (%)	mIoU (%)
64	24.65	40.98	93.42	71.14
128	25.31	41.48	95.34	76.63
256	27.81	43.15	95.53	77.47

**Table 4:** Cityscapes validation set comparison (IoU per category). TOSQ outperforms Trans4Trans in 14/19 classes. Bold numbers indicate the best performance among the compared methods.

Category	Trans4Trans (%)	TOSQ (%)
road	97.04	96.04
sidewalk	71.78	**76.67**
building	86.44	**87.79**
wall	46.24	**48.99**
fence	31.30	**44.31**
pole	39.50	**40.69**
traffic light	**42.49**	22.40
traffic sign	53.62	**60.14**
vegetation	**88.38**	88.08
terrain	50.97	**60.53**
sky	**92.61**	89.57
person	66.14	**67.01**
rider	41.41	**46.10**
car	88.23	**89.60**
truck	50.30	**68.65**
bus	60.00	**74.19**
train	**57.18**	53.54
motorcycle	28.90	**38.26**
bicycle	**58.95**	55.66
mIoU	60.61	**63.59**

## Data Availability

No new data were created or analyzed in this study. Data sharing is not applicable.
